# Economic Cost Analysis of *Acanthamoeba* Keratitis Among Contact Lens Wearers

**DOI:** 10.1097/ICL.0000000000001216

**Published:** 2025-08-15

**Authors:** Dereje Hayilu Anbesse, Stephanie Yeo, Bryan Chong, Blake Angell, Fiona Stapleton, Constantinos Petsoglou, Nicole Ann Carnt

**Affiliations:** School of Optometry and Vision Science (D.H.A., S.Y., B.C., F.S., N.A.C.), UNSW, Sydney, NSW, Australia; George Institute for Global Health (B.A.), and Faculty of Medicine and Health, UNSW, Sydney, NSW, Australia; and Sydney Eye Hospital (C.P.), University of Sydney, Sydney, NSW, Australia.

**Keywords:** Economic cost, *Acanthamoeba* keratitis, Direct costs, Indirect costs

## Abstract

**Objectives:**

This study aimed to estimate direct and indirect economic costs associated with *Acanthamoeba* keratitis and explore variations in individual and healthcare characteristics.

**Methods:**

Patients treated at Moorfields Eye Hospital, UK, between January 2011 and August 2014 were surveyed. Patients were included if they had a confirmed diagnosis and wore contact lenses. Direct costs included healthcare expenses, whereas indirect costs encompassed lost wages for patients and caregivers. The Mann–Whitney *U* test and generalized linear models were used to analyze cost data and their associated factors, with *P*<0.05 being considered statistically significant.

**Results:**

A total of 73 patients were included, with a mean age of 39.3 years (19–81); 40 patients (54.8%) were female, and 41 (56.2%) had good outcomes. Median direct, indirect, and total costs were £2,966.5 (IQR £5,553.2), £1,776.0 (IQR £3,223.3), and £6,678.6 (IQR £17,268.4), respectively. Higher direct costs were independently associated with multiple medical visits (*P*<0.001), steroids before antiamoeba therapy (*P*=0.042), and keratoplasty (*P*<0.001).

**Conclusions:**

The cost of managing *Acanthamoeba* keratitis is substantial. Future research should focus on optimizing treatment strategies and improving patient outcomes to help alleviate the financial burden on patients and healthcare systems.

*Acanthamoeba* keratitis (AK) is a rare and severe corneal infection that most commonly occurs as a complication of contact lens use in high-income countries.^1^ It represents about 2% of all microbial keratitis cases, with outbreaks often resulting from inadequate contact lens hygiene^2^ and environmental factors such as floods.^3,4^ Globally, over 140 million people wear contact lenses, and it is estimated that the annual incidence of AK in this group varies between 17 and 70 per million.^3^ AK cases unrelated to contact lens wearing are also increasing, particularly in low- and middle-income countries in association with exposure to contaminated water, soil, and ocular trauma.^5^

Patients with AK can develop severe ocular signs and symptoms leading to significant visual impairment.^6^ Patient visual outcomes are influenced by the severity of the disease at presentation, delay in diagnosis and treatment, corticosteroid use before diagnosis, and the presence of endosymbionts in *Acanthamoeba* eye isolates.^7–9^ AK severely impacts the quality of life, mainly for patients with delayed diagnosis and severe clinical status.^10^

AK can impose significant financial burdens on society and the health system.^11^ Direct, indirect, and intangible costs associated with AK have not been fully explored, and key cost drivers and the economic burden on patients remain poorly understood. Understanding these elements is crucial for improving healthcare systems, ensuring access to appropriate models of care, and strengthening prevention efforts. Furthermore, it is essential to enhance practitioner awareness to enable prompt diagnosis and treatment.

The study aimed to investigate the direct and indirect costs associated with managing AK in a quaternary hospital in the United Kingdom. Direct costs included expenses related to medical consultations, hospital admission, diagnosis, medications, and surgical procedures, and indirect costs encompassed the economic impact of time off work by affected individuals and caregivers.^12^

We conducted a cost assessment of patients with AK treated between January 2011 and August 2014. The study was conducted from March to September 2024. All expense categories were adjusted to 2024 values using a conversion factor 1.38 (2012 → 2024) derived from the Health Consumer Price Index published by the Bank of England.^13^

## METHODS

### Study Setting and Population

Patients with AK who wore contact lenses and were treated at Moorfields Eye Hospital in London between January 2011 and August 2014 were included in the study. Moorfields Eye Hospital is a quaternary center specializing in treating eye disease, training ophthalmic professionals, and conducting biomedical research for over 200 years. AK was diagnosed based on at least one of the following criteria: a positive corneal biopsy or scrape culture, positive in vivo confocal microscopy, or a typical AK clinical course that responds to antiamoeba therapies. Antiamoebic therapies used in this study included polyhexamethylene biguanide (PHMB) and/or chlorhexidine. These were sometimes combined with diamidine agents, such as propamidine or hexamidine. Other antiamoebic medications reported in previous studies were also considered.^2,14^

Participants were included if they had complete data from the first medical visit to the discharge date, including records of best-corrected visual acuity on the day of discharge or transfer. All study participants were over 18 years old and provided written informed consent for the use of their medical records for research purposes. Ethical approval was obtained from the London and Whittington NHS Ethics Committee (ethics number: 13 LO 0032). The study was conducted in adherence to the tenets of the Helsinki Declaration.

### Data Collection

A self-administered questionnaire was presented to the participants via Opinio software (Opinio; Objectplanet, Oslo, Norway) to collect sociodemographic data, including age, sex, and the last 12 months' salary, while the patients were undergoing treatment. The cost analysis was performed using protocol costing,^15^ relying on published estimates of the national average unit expenditures from the National Schedule of Reference Costs 2011 to 2012 for NHS trusts and the NHS Foundation,^16^ which provided estimates for direct cost data, including expenditures for clinical consultations, admissions, investigations, biopsies, microbiology, and surgical treatments. The prices of relevant pharmaceuticals were extracted from published government estimates provided in the Prescription Cost Analysis for England, 2012.^17^

Indirect costs were assessed by evaluating the lost daily wages of participants and caregivers.

The daily wages of participants were calculated by multiplying the number of days off work, which included medical visits and hospital bed days, by their self-reported average daily income.

The lost wages by caregivers were also estimated by first calculating their days off work associated with medical visits for participants who had more than five medical visits, using a weight of 0.1 days for each visit based on a previous population-based study conducted on the morbidity of contact lens–related microbial keratitis.^11^ These days off work were then added to the total number of hospital bed days experienced by the participants, assuming that caregivers were present with them throughout their hospital stays. The resulting sum was multiplied by the average daily wage to estimate the total lost income.

### Operational Definitions

For this cost study, the disease result was categorized into good and poor outcome groups. For a participant with a history of normal vision, poor outcomes were defined based on the criteria of factors affecting the morbidity of contact lens–related microbial keratitis: a population study, greater than or equal fifth percentile of the sample (in this study 12.7 months or more) and the final visual acuity equal to or worse than 6/24.^11^ All other cases were grouped as having good outcomes. Based on Tu et al.,^9^ and Robaei et al.,^14^ disease stages at presentation were categorized into three stages: Stage one was defined as corneal epitheliopathy alone. Stage two required the presence of any corneal epithelial defect, perineural infiltrate, or stromal infiltrate in addition to corneal epitheliopathy. Stage three encompassed corneal ring infiltrate and at least one feature of stage two characteristics.

### Statistical Analysis

Measures of central tendency (mean and median) and dispersion (SD and interquartile range (IQR) were used to summarize the continuous variables, whereas frequency and percentage were used for categorical variables. Continuous variables such as age and cost data were tested for normality by the Shapiro–Wilk and Kolmogorov–Smirnov tests and were not normally distributed. Therefore, median and IQR were used throughout the analysis, although mean costs were also reported in some cases for comparison with other studies. Cost categories were compared between good and poor outcomes using the Mann–Whitney *U* test. The factors associated with direct cost were examined using a generalized linear model with gamma distribution and log link: an approach best established for right-skewed cost data.^18^ A coefficient greater than zero indicates a positive association between the variables and increased disease costs. For interpretation purposes, the coefficients were converted to percentages using the formula: (e ^coefficient^_-_ 1) x 100. A *P*<0.05 was used to determine statistical significance. The statistical analysis was performed using IBM SPSS Statistics for Windows, Version 27.0. Armonk, NY: IBM Corp.

## RESULTS

A total of 73 cases with confirmed AK diagnosis were included in the study, with a mean age of 39.3 and SD of 16.2 years, and 40 (54.8%) of these were female. Among the cases, 41 had good outcomes, with a mean age of 36.1 (SD 14.1) years, whereas 32 had poor outcomes, with a mean age of 43.5 (SD 18.0) years. Of the cases with good outcomes, 27 (65.9%) were female, whereas in poor outcomes, 13 (40.6%) were female. Of the total, 39 cases (53.4%) were referred to Moorfields Eye Hospital from various eye health sectors. The most common symptom was pain, 56 (75.5%), followed by photophobia, 23 (31.5%).

Among cases with good outcomes, 15 (93.8%) patients were correctly diagnosed with AK, whereas 10 (52.6%) were misdiagnosed with herpetic keratitis. In contrast, among cases with poor outcomes, only one case was correctly diagnosed with AK, and 9 (47.4%) were incorrectly diagnosed as herpetic keratitis (*P*=0.005). All the cases with corneal perforation (n=7) and most scleritis cases (14/17, 82.4%) were classified as poor outcomes. All 17 corneal grafts and all 15 other ocular surgeries occurred in the poor outcome group (Table 1).

**TABLE 1.** Demographic and Clinical Presentations of AK

Variables	Overall	Poor	Good	*P*
Age (mean/SD)	39.3 (16.2)	43.5 (18.0)	36.1 (14.1)	0.19
Sex (%)				0.028
Female	40 (54.8)	13 (40.6)	27 (65.9)	
Male	33 (45.2	19 (59.4)	14 (34.1)	
Ways of presentation (%)				0.057
Self	33 (45.2)	10 (30.3)	23 (69.7)	
Referral	39 (53.4)	22 (56.4)	17 (43.6)	
Affected eye (%)				
Both	3 (4.1)	1 (33.3)	2 (66.7)	
Left eye	41 (56.2)	18 (43.9)	23 (56.1)	
Right eye	29 (39.7)	13 (44.8)	16 (55.2)	
Symptoms (%)				0.28
Pain	56 (75.5)	23 (43.8)	32 (56.2)	
Photophobia	23 (31.5)	10 (43.5)	13 (56.5)	
Lacrimation	13 (17.8)	4 (30.8)	9 (69.2)	
Others	14 (19.2)	4 (28.6)	10 (71.4)	
Duration of symptoms before presentation in months				
Median (IQR)	1.3 (1.1)	1.4 (1.2)	1.2 (1.0)	0.83
Initial diagnosis (%)				0.005
AK	16 (21.9)	1 (6.3)	15 (93.8)	
Bacterial	6 (8.2)	5 (83.3)	1 (16.7)	
Herpetic	19 (26.0)	9 (47.4)	10 (52.6)	
Other complications	22 (30.1)	13 (59.1)	9 (40.9)	
Unknown	5 (6.8)	3 (60.0)	2 (40.0)	
Stage of presentation (%)				0.022
1	17 (23.3)	5 (29.4)	12 (70.6)	
2	35 (47.9)	12 (34.3)	23 (65.7)	
3	8 (11.0)	5 (62.5)	3 (37.5)	
Severe clinical features (%)				
Corneal perforations	7 (9.6)	7 (100)	0	
Scleritis	17 (23.3)	14 (83.4)	3 (16.6)	
Ring infiltrates	22 (30.1)	17 (77.3)	5 (22.7)	
Undergone keratoplasty	17 (23.3)	17 (100)	0	
Other surgeries, except for biopsy	15 (20.5)	15 (100)	0	
Was the patient admitted? (%)				<0.001
No	45 (61.8)	7 (21.9)	38 (92.7)	
Yes	25 (34.2)	23 (71.9)	2 (4.9)	

AK, *Acanthamoeba* Keratitis.

Regarding the pharmaceuticals prescribed, all patients were treated with antiamoeba therapy, such as PHMB and/or chlorhexidine with or without diamidine agents such as propamidine or hexamidine, and other antiamoebic medications. In addition, 29 (39.7%) received steroids and/or nonsteroidal anti-inflammatory drugs after they commenced antiamoeba therapies, and 49 patients (67.1%) were given both topical and oral antibiotics. A total of 68 patients (93.2%) underwent diagnostic corneal scraping during outpatient treatment. Of the 17 corneal grafts performed, 13 (76.5%) were penetrating keratoplasties, three (17.6%) were deep anterior lamellar keratoplasties, and one (5.9%) was unspecified. There were 11 therapeutic keratoplasties (nine tectonic and two for infection control) and five optical keratoplasties for vision correction. Seven patients (9.6%) underwent unilateral cataract extraction in the AK-affected eye, and three (4.1%) had glaucoma surgeries because of AK-related glaucoma.

The discharge assessment and treatment duration were recorded for the good and poor outcomes groups and are presented in Table 2. The median duration of treatment in the good outcomes group was 133.0 (IQR 211.0) days and 390.5 (IQR 670.5) days in poor outcomes, with a significant difference between the two groups (*P*<0.001). Cases with good outcomes had a median of nine medical visits (IQR 12.8), whereas those with poor outcomes had a 26.0 median (IQR 55.0), with a significant difference observed (*P*<0.001). A total of 37 cases (84.1%) with good outcomes and seven cases (15.9%) with poor outcomes were discharged. Four cases (21.1%) with good outcomes and 15 cases (78.9%) with poor outcomes missed their scheduled clinic appointments while data collection was ongoing. Consequently, their final visual outcomes were not recorded in this analysis (Table 2).

**TABLE 2.** Final Medical Visit Outcome Status of Study Participants

Type of Outcome	Overall	Poor	Good	*P*
Final day assessment (median/IQR)				
Duration of treatment in days	200.0 (356.5)	390.5 (670.5)	133.0 (211.0)	<0.001
Number of medical visits	12.0 (24.0)	26.0 (55.0)	9.0 (12.8)	<0.001
Elapsed from symptoms initiation to discharge/loss to follow-up (in months)	12.0 (21.0)	23.0 (34.8)	8.0 (12.0)	0.016
Final day patient status (count/%)				<0.001
Discharged	44 (60.3)	7 (15.9)	37 (84.1)	
Lost to follow-up	19 (26.0)	15 (78.9)	4 (21.1)	
Transferred	5 (6.8)	5 (100)	0	

**FIG. 1.**
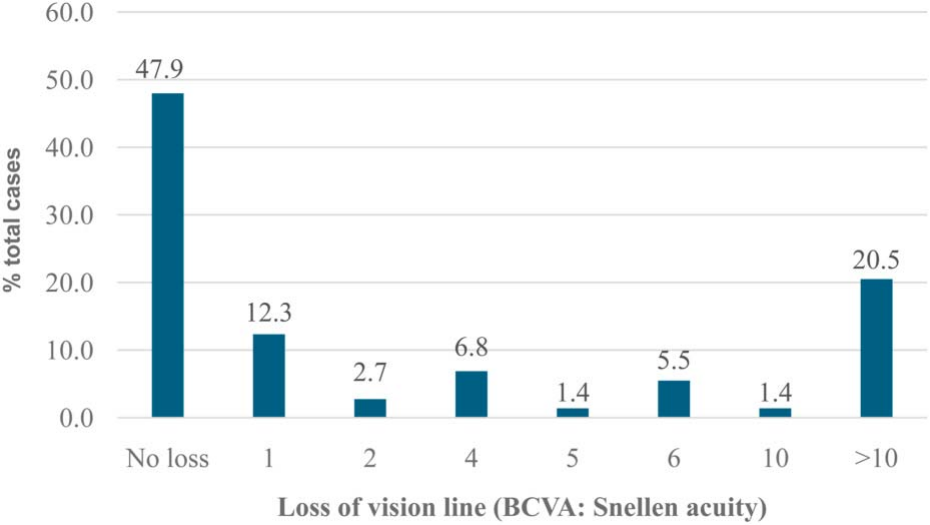
Number of visual acuity lines lost among study participants compared with baseline.

Twenty percent of the cases experienced a loss of more than 10 lines in best-corrected vision after treatment, as measured using the Snellen acuity chart. These cases were classified as monocularly blind based on the definition of blindness of distance visual acuity being worse than 3/60.^19^ Conversely, 47.9% of patients maintained a vision of 6/6 with no loss, whereas 38.3% experienced a loss of at least two lines from their baseline 6/6 acuity (Fig. 1).

The mean total direct cost was £7,277.3 (SD £8,226.7) (mean cost reported here to compare with other studies), and the median was £2,966.5 (IQR £5,553.2). The median for medical visits was £1,360.7 (IQR £1,656.0), accounting for 27.1% of the total direct expenditures. The lowest cost was associated with pharmaceuticals, with a median £125.1 (IQR £251.1), contributing 4.0% of the total direct costs. Hospital bed days, including admission fees, had a median £3,324.4 (IQR £2,398.8), representing 28.4% of the total expenditures (Table 3).

**TABLE 3.** Categories of Direct Costs

Cost Category		Median (IQR)	Mean (SD)	Min-max	% Total
Direct costs (n=73)	Medical visits	1,360.7 (1,656.0)	2,322.1 (2,547.4)	422.3–13,283.9	27.1%
	Hospital bed days and admission	3,324.4 (2,398.8)	2,433.9 (1,312.2)	590.6–3,324.4	28.4%
	Investigations	788.0	839.5 (437.5)	45.5–2,272.9	9.8%
	Pharmaceuticals	125.1 (251.1)	337.6 (545.3)	9.67–2,832.2	4.0%
	Surgical interventions	742.4 (865.9)	2,628.3 (3,811.2)	742.4–17,503.9	30.7%
	Total	2,966.5 (5,553.2)	7,277.3 (8,226.7)	1,556.2, 36,908.0	100%

The mean cost reported here is to compare values with other studies.

The highest median cost was for daily wage loss by participants, which was £1,545.6 (IQR £2,898.0), accounting for 89.9% of the total indirect loss. The median loss by caregivers was £161.5 (IQR £318.1). The median for all indirect expenditures was £1,776.0 (IQR £3,223.3; Table 4).

**TABLE 4.** Categories of Indirect Costs

Cost Category		Median (IQR)	Mean	Min-max	% Total Cost
Indirect cost (N=47)	Daily wage loss	1,545.6 (2,898.0)	3,355.7	269.1–22,245.6	89.9
	Caregivers lost	161.5 (318.1)	376.7	26.9–2,296.3	10.1
	Total	1,776.0 (3,223.3)	3,920.4	296.0–24.541.9.2	100

Note: The mean cost reported here is to compare values with other studies.

**FIG. 2.**
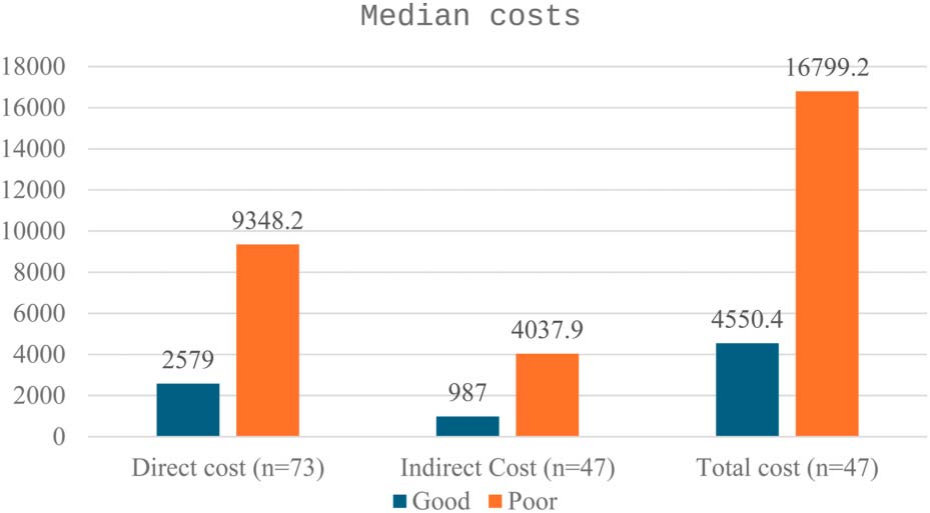
Direct and indirect costs between good and poor outcomes.

There were significantly higher costs in the poor outcomes group. The median direct costs were £2,579.0 in the good outcomes group and £9,348.2 in the poor outcomes group. Indirect costs were £987.0 for the good outcomes group and £4,037.9 for the poor outcomes group. The median costs were £4,550.4 in the good and £16,799.2 in the poor outcomes group. Overall, the median total costs were £6,678.6 (IQR £17,268.4), with 65.8% attributed to direct costs and 34.2% to indirect costs (Fig. 2).

The median costs associated with medical visits, pharmaceuticals, surgical procedures, and overall and indirect costs varied significantly between cases with good and poor outcomes (*P*<0.001) when comparing different cost categories. However, there were no significant variations in the expenses for hospital bed days, admission, and investigations (*P*=0.721 and 0.527, respectively; Table 5).

**TABLE 5.** Comparison of Direct and Indirect Costs

Cost types	Poor	Good	Mann–Whitney U	*P*
	Median	Median		
Direct costs				
Medical visits	2,795.9	1,029.5	175.5	<0.001
Hospital bed days and admission	3,324.4	2,103.8	20.0	0.721
Investigations	788.0	788.0	606.5	0.527
Pharmaceuticals	276.9	57.5	187.0	<0.001
Surgical interventions	4,206.2	742.4	273.0	<0.001
Total direct	9,348.2	2,579.0	188.0	<0.001
Indirect cost				
Total daily wage and caregivers	4,037.9	987.0	129.0	0.002

In the univariable analysis, higher direct costs were positively associated with variables such as initial misdiagnosis (*P*<0.001), stage two and three at diagnosis (*P*=0.043), frequent medical visits (*P*<0.001), longer duration of antiamoeba therapy in months (*P*<0.001), use of steroids before antiamoeba therapy (*P*=0.005), and keratoplasty surgery (*P*<0.001). In multivariable generalized linear model analysis, frequent medical visits (*P*<0.001), using steroids before antiamoeba therapy (*P*=0.037), and keratoplasty surgery (*P*<0.001) were identified as independent risk factors associated with higher direct costs. The direct costs increased by 1.6% for each additional medical visit, 7.5% for steroid use before antiamoeba therapy, and 199.8% for each keratoplasty surgery (Table 6).

**TABLE 6.** Factors Associated With direct Cost Using a Generalized Linear Model

Variables	Direct Cost			
	Univariable β (95 CI)	*P*	Multivariable β (95 CI)	*P*
Age inverse	0.004 (−0.009 to 0.016)	0.574		
Female sex	0.304 (−0.103 to 0.711)	0.144		
Initially misdiagnosed	1.145 (0.697–1.593)	<0.001		
Severe disease stage	0.525 (0.017–1.033)	0.043		
Number of follow-up medical visits	0.034 (0.027–0.042)	<0.001	0.016 (0.011–0.022)	<0.001
Duration of antiamoeba therapy in months	0.043 (0.024–0.062)	<0.001		
Use of steroids before antiamoeba therapy	0.690 (0.206–1.174)	0.005	0.072 (0.004–0.14)	0.037
Keratoplasty	1.726 (1.464–1.989)	<0.001	1.098 (0.855–1.341)	<0.001

## DISCUSSION

This study is the first to examine the economic burden of patients with AK and the healthcare system. The financial burden associated with AK treatment is considerably higher than that of other microbial keratitis, reflecting the complexity and intensity of its management.

The median direct cost per patient was £2,966.5, with surgical and procedural expenses combined with hospital bed days accounting for 59.1% of total direct costs. Although we found limited existing economic studies examining the cost of treatment for patients with AK, these figures are notably higher than those in other reported microbial keratitis studies, where the average treatment costs for patients were about £1,300 in two previous studies.^20,21^ However, our findings align with a 2013 UK study on contact lens–related microbial keratitis, which reported a median cost of £2,855. The higher costs in UK-based studies may reflect not only disease severity but also NHS costing methods, which capture a broader range of healthcare resources compared with insurance claims or hospital charges used in former studies.

Hospital admissions and bed days accounted for a large component of the total expenses in our study. This is consistent with the findings of Daley et al., who reported that the admission costs were by far the most significant contributor, accounting for 71% of the direct medical costs.^22^ These findings have important implications for both clinical practice and health policy. The substantial economic burden of inpatient care highlights the need for earlier diagnosis and more effective outpatient management strategies to reduce hospital admissions.

Our study found the median total indirect cost was £1,776.00, which is higher than those reported for contact lens–related microbial keratitis, such as £468.00 in the Australian,^11^ and £245.30 (USD314.50) in the Taiwan study.^23^ Although the latter study identified indirect costs as the primary cost driver, our findings revealed a contrasting pattern, with 65.8% of total costs attributable to direct healthcare expenditures.

The median total cost for managing AK in our study was £6,678.60. This amount significantly exceeds the reported costs for microbial keratitis in the United States (£2,645.31).^20^ Keay et al.^24^ reported that the treatment costs for contact lens–related microbial keratitis varied by severity: £683.09 (AUD 1,596) for severe cases with vision loss, £530.81 (AUD 795) for mild cases, and a similar cost for severe cases without vision loss. Another study from northern China reported that the median medical costs of viral, fungal, and bacterial keratitis with inpatient care were £811.50 (USD867), £961.27 (USD1,027), and £731.95 (USD782), respectively. The variation observed here might be because medical expenditure in each country varied based on the availability of medical resources, economic level, and health system structures.

Our findings revealed that the costs of AK treatment are generally greater than another microbial keratitis. Although direct comparisons are limited, this trend of increased costs for more severe or difficult-to-treat infections is consistent with an Indian study,^25^ which found that treatment costs for fungal keratitis, typically more complex than bacterial keratitis, were also higher. This is likely because of the higher frequency of medical consultations and hospitalizations required by severe eye infections, reflecting the persistent and difficult-to-treat nature of the infection.^26^ In AK, delayed diagnosis and limited availability of effective antiamoebic treatments complicate management.^6^ Currently, there is no licensed single-agent topical therapy for AK treatment, and up to 30% of patients require surgical intervention, such as corneal transplantation.^3^

Several factors were significantly associated with higher direct costs. Direct expenditures increased by 1.61% for each additional medical visit. The need for frequent surgical treatment also poses a barrier to achieving favorable outcomes, requiring extensive follow-ups and longer courses of care.^27^ Another two studies reported that longer stays in the hospital were associated with high costs of microbial keratitis.^23,28^

Furthermore, direct costs increased by 7.48% with prior steroid use before antiamoeba therapy. Steroid use before antiamoeba therapy exacerbates disease severity and delays diagnosis, leading to poorer outcomes and increased financial burden. Caution should, therefore, be exercised with steroid use, emphasizing the need for early and accurate diagnosis.^29,30^

Direct costs were increased by 199.8% after each keratoplasty procedure. Among 17 patients who underwent keratoplasty, the mean duration of antiamoeba treatment was 555.9 days, with most requiring additional procedures. This finding is consistent with a previous study in China, which reported that keratoplasty was the key driver for the medical costs of infectious keratitis. Keratoplasty is a complex and prolonged treatment course that imposes significant economic burdens, consistent with other studies highlighting how procedural costs and frequent medical visits exacerbate the financial strain.^20,31^

The economic burden of AK is expected to vary significantly across regions and healthcare systems, similar to that of microbial keratitis. In countries with less structured healthcare systems and lower income levels, AK poses a considerable societal and financial burden.^1,11,23^ Out-of-pocket costs may be substantially higher in these settings and in health systems where patients pay for nonsubsidized treatments and medication.^27^ Despite its rarity, AK represents a severe corneal emergency with challenging treatment implications.^27,32,33^ The high financial cost of managing AK underscores the urgent need for preventive strategies and investments in diagnostics and therapies that can reduce the need for frequent visits and invasive procedures, such as keratoplasty.

This study has several limitations. First, the retrospective nature of cost data collection may potentially overlook specific expenses. For instance, travel costs for patients and caregivers were not included because of data limitations. In addition, the cost data used was more than 10 years old. Although we adjusted the figures for inflation to reflect 2024 values, this may not fully account for changes in clinical practice, healthcare delivery, or financial structures that have occurred over time.

## CONCLUSION

A significant proportion of contact lens wearers with AK experience moderate to severe visual impairment. The economic burden of the diseases is substantial, with direct costs comprising most of the total expenses. The key factors associated with higher direct costs include the number of medical visits, prior use of steroids before antiamoeba therapy, and the necessity for keratoplasty surgeries. To support informed decision-making and enhance understanding of the economic impact, future research should include a comprehensive cost-of-illness analysis that accounts for medical, nonmedical, and intangible expenses, along with an evaluation of the cost-effectiveness within the healthcare system. It should also focus on optimizing treatment strategies and improving patient outcomes to help alleviate the financial burden on patients and healthcare systems.

## References

[1] Stapleton F. The epidemiology of infectious keratitis. Ocul Surf 2023;28:351–363. 34419639 10.1016/j.jtos.2021.08.007

[2] Carnt N Hoffman JJ Verma S . Acanthamoeba keratitis: Confirmation of the UK outbreak and a prospective case-control study identifying contributing risk factors. Br J Ophthalmol 2018;102:1621–1628. 30232172 10.1136/bjophthalmol-2018-312544

[3] Rayamajhee B Willcox MD Henriquez FL . Acanthamoeba keratitis: An increasingly common infectious disease of the cornea. Lancet Microbe 2021;2:e345–e346. 35544193 10.1016/S2666-5247(21)00093-8

[4] Höllhumer R Keay L Watson SL. Acanthamoeba keratitis in Australia: Demographics, associated factors, presentation and outcomes: A 15-year case review. Eye 2020;34:725–732. 31554948 10.1038/s41433-019-0589-6 PMC7093427

[5] Ilyas M Stapleton F Willcox MDP . Epidemiology of and genetic factors associated with Acanthamoeba keratitis. Pathogens 2024;13:142. 38392880 10.3390/pathogens13020142 PMC10892102

[6] Dart JK Saw VP Kilvington S. Acanthamoeba keratitis: Diagnosis and treatment update 2009. Am J Ophthalmol 2009;148:487–499.e2. 19660733 10.1016/j.ajo.2009.06.009

[7] Carnt N Robaei D Minassian DC . Acanthamoeba keratitis in 194 patients: Risk factors for bad outcomes and severe inflammatory complications. Br J Ophthalmol 2018;102:1431–1435. 29298778 10.1136/bjophthalmol-2017-310806

[8] Rayamajhee B Sharma S Willcox M . Assessment of genotypes, endosymbionts and clinical characteristics of Acanthamoeba recovered from ocular infection. BMC Infect Dis 2022;22:757. 36175838 10.1186/s12879-022-07741-4 PMC9520893

[9] Tu EY Joslin CE Sugar J . Prognostic factors affecting visual outcome in Acanthamoeba keratitis. Ophthalmology 2008;115:1998–2003. 18571729 10.1016/j.ophtha.2008.04.038 PMC3816554

[10] Di ZA Varacalli G Coassin M . Acanthamoeba keratitis: Perspectives for patients. Invest Ophthalmol Vis Sci 2020;61:4892.

[11] Keay L Edwards K Naduvilath T . Factors affecting the morbidity of contact lens-related microbial keratitis: A population study. Invest Ophthalmol Vis Sci 2006;47:4302–4308. 17003419 10.1167/iovs.06-0564

[12] Angevine PD Berven S. Health economic studies: An introduction to cost-benefit, cost-effectiveness, and cost-utility analyses. Spine (Phila Pa 1976) 2014;39:S9–S15. 25299265 10.1097/BRS.0000000000000576

[13] England Bo. Infilation calculator. 2024 [cited 2024 February 15, 2024]; Available at: bankofengland.co.uk.

[14] Robaei D Carnt N Minassian DC . Therapeutic and optical keratoplasty in the management of Acanthamoeba keratitis: Risk factors, outcomes, and summary of the literature. Ophthalmology 2015;122:17–24. 25262318 10.1016/j.ophtha.2014.07.052

[15] Jackson T. Cost estimates for hospital inpatient care in Australia: Evaluation of alternative sources. Aust N Z J Public Health 2000;24:234–241. 10937398 10.1111/j.1467-842x.2000.tb01562.x

[16] Department of Health. National Schedule of Reference Costs: 2011–12 for NHS Trusts and NHS Foundation Trusts. London: Department of Health; Available from: https://www.gov.uk/government/publications/nhs-reference-costs-2011-to-2012. (2011–2012).

[17] England N, editor. Prescription Cost Analysis - England, 2012. London: NHS Digital (Health and Social Care Information Centre); Available from: https://digital.nhs.uk/data-and-information/publications/statistical/prescription-cost-analysis/prescription-cost-analysis-england-2012 (2012).

[18] Mohammadpour S Niknam N Javan-Noughabi J . The factors associated with direct medical costs in patients with gastric cancer: Quantile regression approach compared with gamma regression. Value Health Reg Issues 2020;21:127–132. 31783308 10.1016/j.vhri.2019.08.479

[19] WHO. World Report on Vision. Geneva: WHO; 2019.

[20] Ashfaq H Maganti N Ballouz D . Procedures, visits, and procedure costs in the management of microbial keratitis. Cornea 2021;40:472–476. 33214415 10.1097/ICO.0000000000002534 PMC8062275

[21] Shi Q Peng B Cheng Z . Direct cost analysis of microbial keratitis in North China: A hospital-based retrospective study. Pathogens 2024;13:666. 39204266 10.3390/pathogens13080666 PMC11357569

[22] Daley JR Lee MK Wang X . Epidemiology and economic cost analysis of microbial keratitis from a tertiary referral hospital in Australia. Pathogens 2023;12:413, 36986335 10.3390/pathogens12030413 PMC10059868

[23] Chantra S Jittreprasert S Chotcomwongse P . Estimated direct and indirect health care costs of severe infectious keratitis by cultured organisms in Thailand: An 8-year retrospective study. PLoS One 2023;18:e0288442. 37437049 10.1371/journal.pone.0288442 PMC10337972

[24] Keay L Edwards K Dart J . Grading contact lens-related microbial keratitis: Relevance to disease burden. Optom Vis Sci 2008;85:531–537. 18594345 10.1097/OPX.0b013e31817dba2e

[25] Radhakrishnan N Pathak N Subramanian KR . Comparative study on costs incurred for treatment of patients with bacterial and fungal keratitis - a retrospective analysis. Indian J Ophthalmol 2022;70:1191–1195. 35326013 10.4103/ijo.IJO_2176_21 PMC9240582

[26] Niederkorn JY. The biology of Acanthamoeba keratitis. Exp Eye Res 2021;202:108365. 33221372 10.1016/j.exer.2020.108365 PMC7856181

[27] Varacalli G Di Zazzo A Mori T . Challenges in Acanthamoeba keratitis: A review. J Clin Med 2021;10, 942. 33804353 10.3390/jcm10050942 PMC7957573

[28] Xie H Liu H Wang C Economic burden of keratitis patients with inpatient care: A nationwide, multicenter registry study; 2024. 10.1016/j.jtos.2025.08.007 40840833

[29] Wouters KA Verhoekx JS Van Rooij J . Topical corticosteroids in Acanthamoeba keratitis: Friend or foe? Eur J Ophthalmol 2022;32:170–175. 33183081 10.1177/1120672120973606

[30] Carnt N Robaei D Watson SL . The impact of topical corticosteroids used in conjunction with antiamoebic therapy on the outcome of Acanthamoeba keratitis. Ophthalmology 2016;123:984–990. 26952591 10.1016/j.ophtha.2016.01.020

[31] Hossain P. Microbial keratitis—the true costs of a silent pandemic? Eye 2021;35:2071–2072. 33594242 10.1038/s41433-020-01360-6 PMC8302703

[32] Szentmáry N Goebels S Matoula P . Acanthamoeba keratitis--a rare and often late diagnosed disease. Klinische Monatsblätter für Augenheilkunde 2012;229:521–528. 22592343 10.1055/s-0031-1299539

[33] Vilares-Morgado R Ferreira AM Marques-Couto P . Clinical outcomes and prognostic factors in Acanthamoeba keratitis. Contact Lens Anterior Eye J Br Contact Lens Assoc 2024;47:102119. 10.1016/j.clae.2023.102119 38220498

